# Supplementary material for: PCGF6 controls neuroectoderm specification of human pluripotent stem cells by activating SOX2 expression
Source: Nat Commun. 2022 Aug 6;13:4601. doi: 10.1038/s41467-022-32295-z (PMC9357003; doi:10.1038/s41467-022-32295-z)
Supplement: Supplementary file 1 — Supplementary Information [file 41467_2022_32295_MOESM1_ESM.pdf]

Supplementary Materials for

PCGF6 controls neuroectoderm specification of human pluripotent stem cells by activating SOX2 expression

This PDF file includes:

Supplementary Figures 1 to 5

Supplementary Tables 1 to 2

Index of Supplemental Information

Supplementary Figures

Supplementary Figure 1. Deletion of PCGF6 does not affect pluripotency maintenance of human PSCs.

Supplementary Figure 2. PCGF6 regulates endoderm differentiation of human PSCs.

Supplementary Figure 3. PCGF6 knockout delays pluripotency exits during neuroectoderm differentiation.

Supplementary Figure 4. SRE-deficiency blocks neuroectoderm differentiation of human PSCs.

Supplementary Figure 5. Analyses of the co-occupancy of PCGF6 and MYC.

Supplementary Tables

Supplementary Table 1. Primers for RT-qPCR analyses

Supplementary Table 2. Primers for ChIP-qPCR analyses

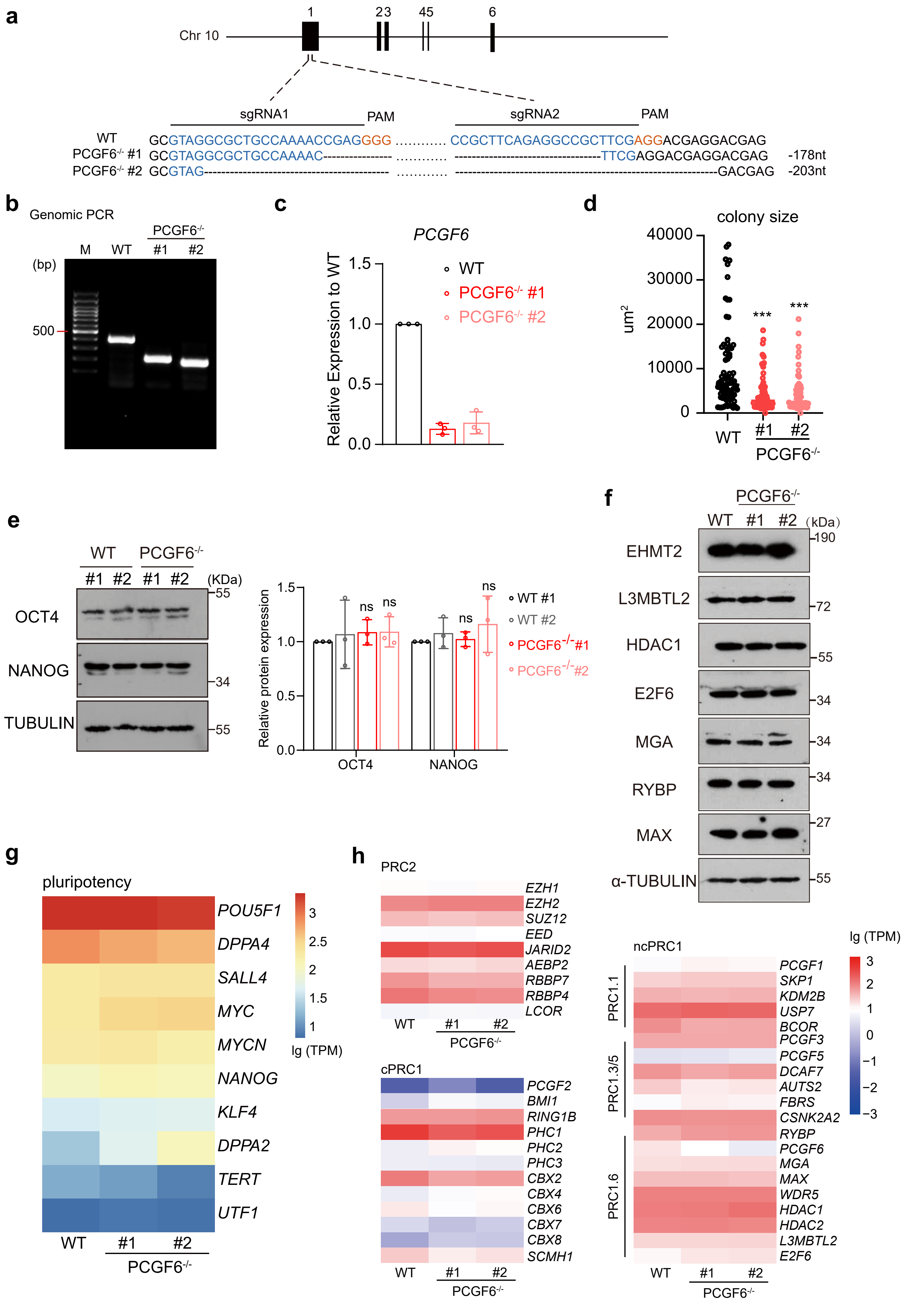
a
Chr 10
1
23
45
6
sgRNA1
PAM
sgRNA2
PAM
WT GCGTAGGCGCTGCCAAAACCGAGGGG .... CCGCTTCAGAGGCCGCTTCGAGGACGAGGACGAG
PCGF6-/- #1 GCGTAGGCGCTGCCAAAAC--------------- .... ---------------------------------TTCGAGGACGAGGACGAG -178nt
PCGF6-/- #2 GCGTAG-- .... --GACGAG -203nt
b
Genomic PCR
PCGF6-/-
M WT #1 #2
(bp)
500
c
PCGF6
Relative Expression to WT
1.5
1.0
0.5
0.0
WT
PCGF6-/- #1
PCGF6-/- #2
d
colony size
um2
40000
30000
20000
10000
0
***
***
WT
#1
#2
PCGF6-/-
e
WT PCGF6-/-
#1 #2 #1 #2
(KDa)
OCT4
NANOG
TUBULIN
55
34
55
Relative protein expression
1.5
1.0
0.5
0.0
ns
ns
ns
ns
OCT4
NANOG
WT #1
WT #2
PCGF6-/-#1
PCGF6-/-#2
f
PCGF6-/-
WT #1 #2 (kDa)
EHMT2
L3MBTL2
HDAC1
E2F6
MGA
RYBP
MAX
α-TUBULIN
190
72
55
34
34
34
27
55
g
pluripotency
POU5F1
DPPA4
SALL4
MYC
MYCN
NANOG
KLF4
DPPA2
TERT
UTF1
3
2.5
2
1.5
1
lg (TPM)
WT #1 #2
PCGF6-/-
h
PRC2
EZH1
EZH2
SUZ12
EED
JARID2
AEBP2
RBBP7
RBBP4
LCOR
WT #1 #2
PCGF6-/-
cPRC1
PCGF2
BMI1
RING1B
PHC1
PHC2
PHC3
CBX2
CBX4
CBX6
CBX7
CBX8
SCMH1
WT #1 #2
PCGF6-/-
ncPRC1
PRC1.1
PRC1.3/5
PRC1.6
PCGF1
SKP1
KDM2B
USP7
BCOR
PCGF3
PCGF5
DCAF7
AUTS2
FBRS
CSNK2A2
RYBP
PCGF6
MGA
MAX
WDR5
HDAC1
HDAC2
L3MBTL2
E2F6
WT #1 #2
PCGF6-/-
lg (TPM)
3
2
1
0
−1
−2
−3

Supplementary Figure 1. Deletion of PCGF6 does not affect pluripotency maintenance of human PSCs.

a. Schematic diagram of guide RNA design targeting PCGF6 and genotypes of generated PCGF6-KO clones. The sgRNA-targeting sequences were highlighted in blue, and PAM sequences are in red. The below schematic diagram showing the genotype of the two PCGF6 knockout clones (178bp and 203bp deletion respectively). **b.** Genotyping analysis of PCGF6 knockout human PSCs by PCR (n = 3). **c.** Relative expression level of *PCGF6* in the wild-type (WT) and PCGF6-KO human PSCs. The level of gene expression in the WT human PSCs is set as 1 (n = 3). **d.** Colony size of WT and PCGF6-KO cells (n = 80). **e.** Protein level of OCT4 and NANOG in WT and PCGF6-KO human PSCs; TUBULIN was used as loading control (n = 3). **f.** Protein level of PRC1.6 components (EHMT2, L3MBTL2, HDAC1, E2F6, MGA, RYBP, MAX) in WT and PCGF6-KO human PSCs; α-TUBULIN was used as loading control (n = 3). **g-h.** Heatmap illustrating the RNA expression in WT and PCGF6-KO human PSCs for selected genes of pluripotency **(g)** and components of PRC complexes (**h)** based on RNA-seq analysis. TPM, Transcripts Per Kilobase Million. Each point represents a biological replicate. Data are presented as the mean ± SD. Statistical significance was determined using the unpaired, two-tailed t-test in **c**, **d**, **e** (*$p<0.05$, **$p<0.01$, ***$p<0.001$). Exact *P* values and statistical parameters are provided as a Source Data file.

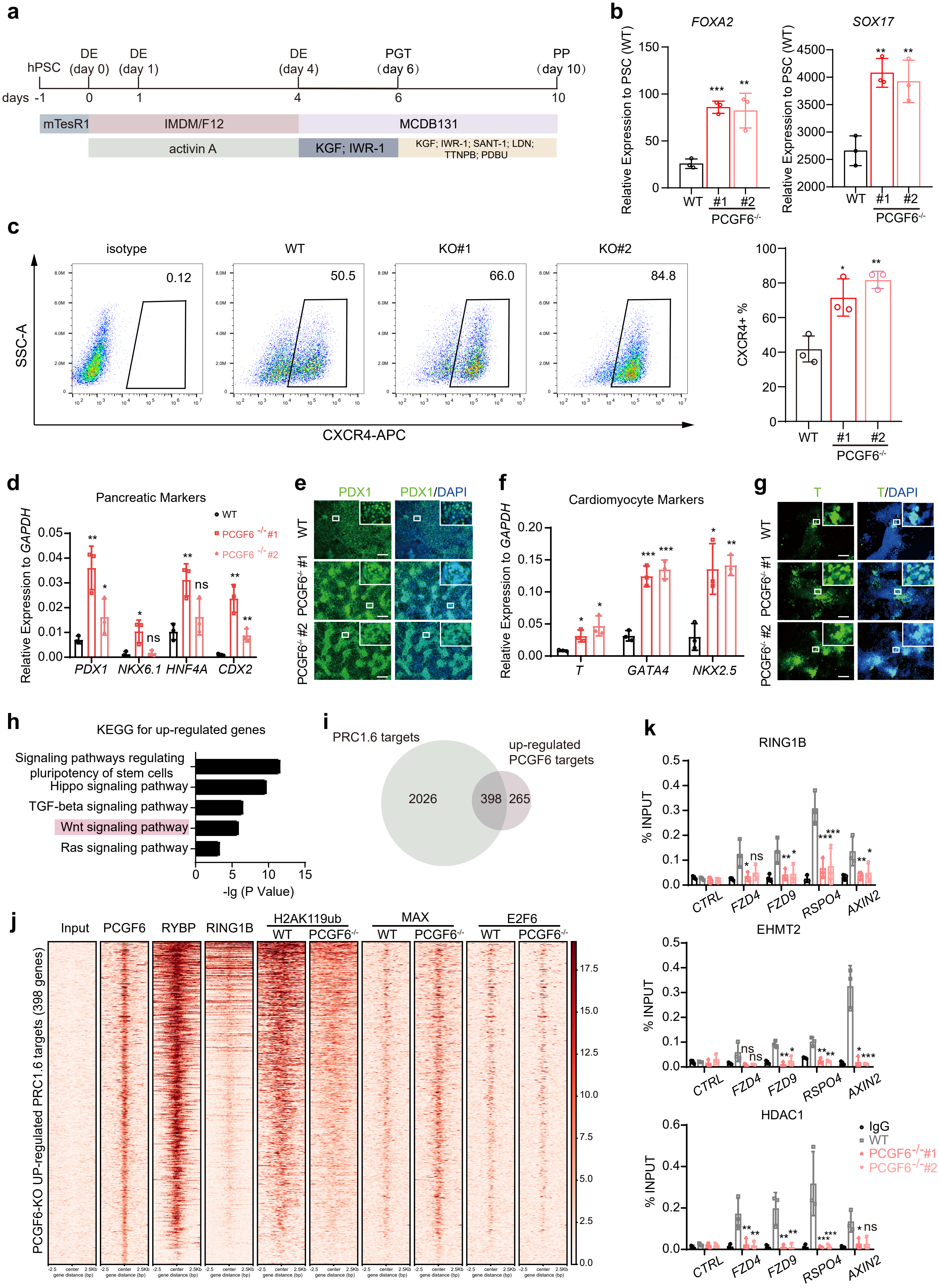

a
hPSC
DE (day 0)
DE (day 1)
DE (day 4)
PGT (day 6)
PP (day 10)
days -1 0 1 4 6 10
mTesR1
IMDM/F12
MCDB131
activin A
KGF; IWR-1
KGF; IWR-1; SANT-1; LDN; TTNPB; PDBU
b
FOXA2
SOX17
Relative Expression to PSC (WT)
WT #1 #2
PCGF6-/-
c
isotype
WT
KO#1
KO#2
0.12
50.5
66.0
84.8
SSC-A
CXCR4-APC
CXCR4+ %
d
Pancreatic Markers
WT
PCGF6 -/- #1
PCGF6 -/- #2
Relative Expression to GAPDH
PDX1 NKX6.1 HNF4A CDX2
e
PDX1
PDX1/DAPI
WT
PCGF6-/- #1
PCGF6-/- #2
f
Cardiomyocyte Markers
Relative Expression to GAPDH
T GATA4 NKX2.5
g
T
T/DAPI
h
KEGG for up-regulated genes
Signaling pathways regulating pluripotency of stem cells
Hippo signaling pathway
TGF-beta signaling pathway
Wnt signaling pathway
Ras signaling pathway
-lg (P Value)
i
PRC1.6 targets
up-regulated PCGF6 targets
2026
398
265
j
Input PCGF6 RYBP RING1B
H2AK119ub
MAX
E2F6
WT PCGF6-/-
PCGF6-KO UP-regulated PRC1.6 targets (398 genes)
gene distance (bp)
k
RING1B
EHMT2
HDAC1
% INPUT
CTRL FZD4 FZD9 RSPO4 AXIN2
IgG
WT
PCGF6-/-#1
PCGF6-/-#2

Supplementary Figure 2. PCGF6 regulates endoderm differentiation of human PSCs.

a. Schematic representation of definitive endoderm (DE) and pancreatic differentiation of human PSCs. **b.** qRT-PCR analysis for expression of endoderm markers (*FOXA2* and *SOX17*) in WT and PCGF6-KO human PSCs during endoderm differentiation. Results are shown relative to undifferentiated WT (n = 3). **c.** Left panel: Flow cytometric analysis showing the percentage of CXCR4-positive cells in differentiated WT and PCGF6-KO cells. Right panel: the percentage of CXCR4-positive cells was counted (n = 3). **d.** qRT-PCR analysis for expression of pancreatic markers (*PDX1, NKX6.1, HNF4A* and *CDX2*) in WT and PCGF6-KO human PSCs during pancreatic differentiation (n = 3). **e.** Immunostaining of the pancreatic marker (PDX1) in WT and PCGF6-KO cells after pancreatic differentiation (n = 3). Scale bars, 200 μm. **f.** qRT-PCR analysis for expression of cardiac markers (*T, GATA4* and *NKX2.5*) in WT and PCGF6-KO human PSCs during cardiac differentiation (n = 3). **g.** Immunostaining of the cardiac marker (T) in WT and PCGF6-KO cells after cardiac differentiation (n = 3). Scale bars, 200 μm. **h.** KEGG pathway analysis of up-regulated genes in PCGF6-KO PSCs. The y axis shows the KEGG signaling pathway categories, the x axis refers to pathway enrichment. **i.** Venn diagram showing the overlap of PRC1.6 targets in WT cells and up-regulated PCGF6 targets in PCGF6-KO cells. **j.** The heatmap showing the enrichment of PCGF6, RYBP, RING1B, MAX (WT and PCGF6-KO), E2F6 (WT and PCGF6-KO) and H2AK119ub (WT and PCGF6-KO) at PCGF6-related PRC1.6 targets in human PSCs. ChIP-seq data of RYBP and RING1B were obtained from GEO database GSM2805870 and GSM2805868, respectively. **k.** ChIP-qPCR analysis for RING1B, EHMT2 and HDAC1 binding at WNT genes (*FZD4*, *FZD9*, *RSPO4* and *AXIN2*) in human PSCs. IgG served as negative control. ChIP enrichments are normalized to Input (n = 3). Each point represents a biological replicate. Data are presented as the mean ± SD. Statistical significance was determined using the unpaired, two-tailed t-test in **b**-**d**, **f**, **k** (*$p<0.05$, **$p<0.01$, ***$p<0.001$). Exact *P* values and statistical parameters are provided as a Source Data file.

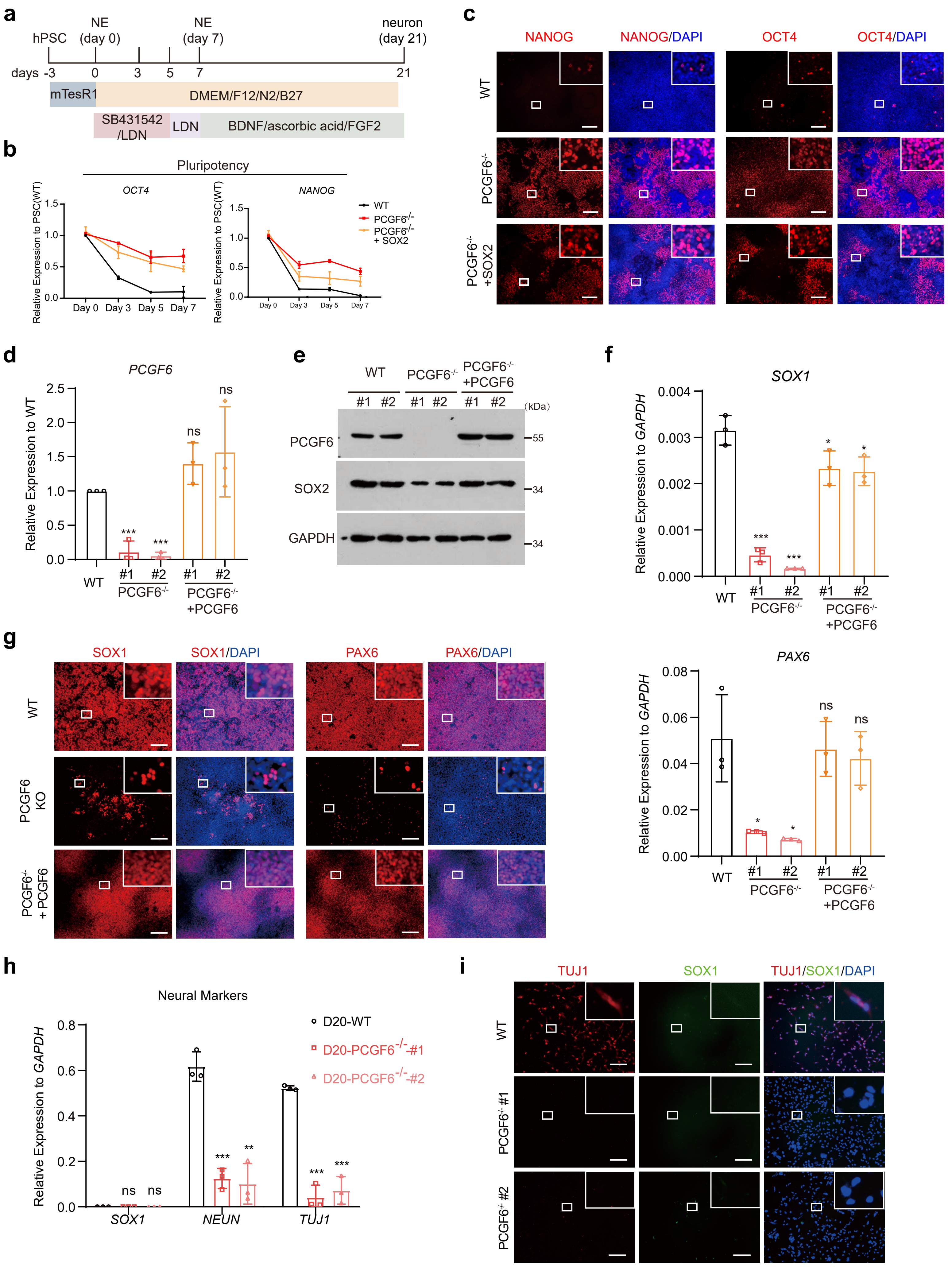
a
hPSC
NE (day 0)
NE (day 7)
neuron (day 21)
days -3 0 3 5 7 21
mTesR1
DMEM/F12/N2/B27
SB431542 /LDN
LDN
BDNF/ascorbic acid/FGF2
b
Pluripotency
OCT4
NANOG
Relative Expression to PSC(WT)
Day 0 Day 3 Day 5 Day 7
WT
PCGF6-/-
PCGF6-/- + SOX2
c
NANOG
NANOG/DAPI
OCT4
OCT4/DAPI
WT
PCGF6-/-
PCGF6-/- +SOX2
d
PCGF6
Relative Expression to WT
WT
#1 #2
PCGF6-/-
PCGF6-/- +PCGF6
ns
***
e
WT
PCGF6-/-
PCGF6-/- +PCGF6
#1 #2 #1 #2 #1 #2
(kDa)
PCGF6
SOX2
GAPDH
55
34
34
f
SOX1
Relative Expression to GAPDH
g
SOX1
SOX1/DAPI
PAX6
PAX6/DAPI
WT
PCGF6 KO
PCGF6-/- + PCGF6
PAX6
h
Neural Markers
Relative Expression to GAPDH
D20-WT
D20-PCGF6-/--#1
D20-PCGF6-/--#2
SOX1
NEUN
TUJ1
i
TUJ1
SOX1
TUJ1/SOX1/DAPI
WT
PCGF6-/- #1
PCGF6-/- #2

Supplementary Figure 3. PCGF6 knockout delays pluripotency exits during neuroectoderm differentiation.

a. Schematic representation of neuronal differentiation of human PSCs. **b.** qRT-PCR analysis for expression of pluripotent markers (*OCT4* and *NANOG*) in WT, PCGF6-KO and SOX2-rescue cells during neuroectoderm differentiation. Results are shown relative to undifferentiated WT (n = 2). **c.** Immunostaining of pluripotent markers (OCT4 and NANOG) in WT, PCGF6-KO and SOX2-rescue cells after neuroectoderm differentiation (n = 3). Scale bars, 200 μm. **d.** qRT-PCR analysis of *PCGF6* expression in WT, PCGF6-KO, $PCGF6^{-/-}$+PCGF6 (PCGF6-KO/PCGF6 re-expressing) human PSCs. Results are shown relative to WT (n = 3). **e.** Protein level of PCGF6 and SOX2 in WT, PCGF6-KO and $PCGF6^{-/-}$+PCGF6 human PSCs was evaluated and quantified; GAPDH was used as a loading control (n = 3). **f.** qRT-PCR analysis for expression of neural markers (*SOX1* and *PAX6*) in WT, PCGF6-KO and $PCGF6^{-/-}$+PCGF6 cells during neuroectoderm differentiation (n = 3). **g.** Immunostaining of the neural progenitor markers (SOX1 and PAX6) in WT, PCGF6-KO and $PCGF6^{-/-}$+PCGF6 cells after neuroectoderm differentiation (n = 3). Scale bars, 200 μm. **h.** qRT-PCR analysis for expression of neural progenitor marker (*SOX1*) and neuron markers (*NEUN* and *TUJ1*) in WT and PCGF6-KO human PSCs after neuron differentiation (21-days) (n = 3). **i.** Immunostaining of neural markers (TUJ1 and SOX1) in WT, and PCGF6-KO cells after neuron differentiation (n = 3). Scale bars, 200 μm. Each point represents a biological replicate. Data are presented as the mean ± SD. Statistical significance was determined using the unpaired, two-tailed t-test in **b**, **d**, **f**, **h** (*$p<0.05$, **$p<0.01$, ***$p<0.001$). Exact *P* values and statistical parameters are provided as a Source Data file.

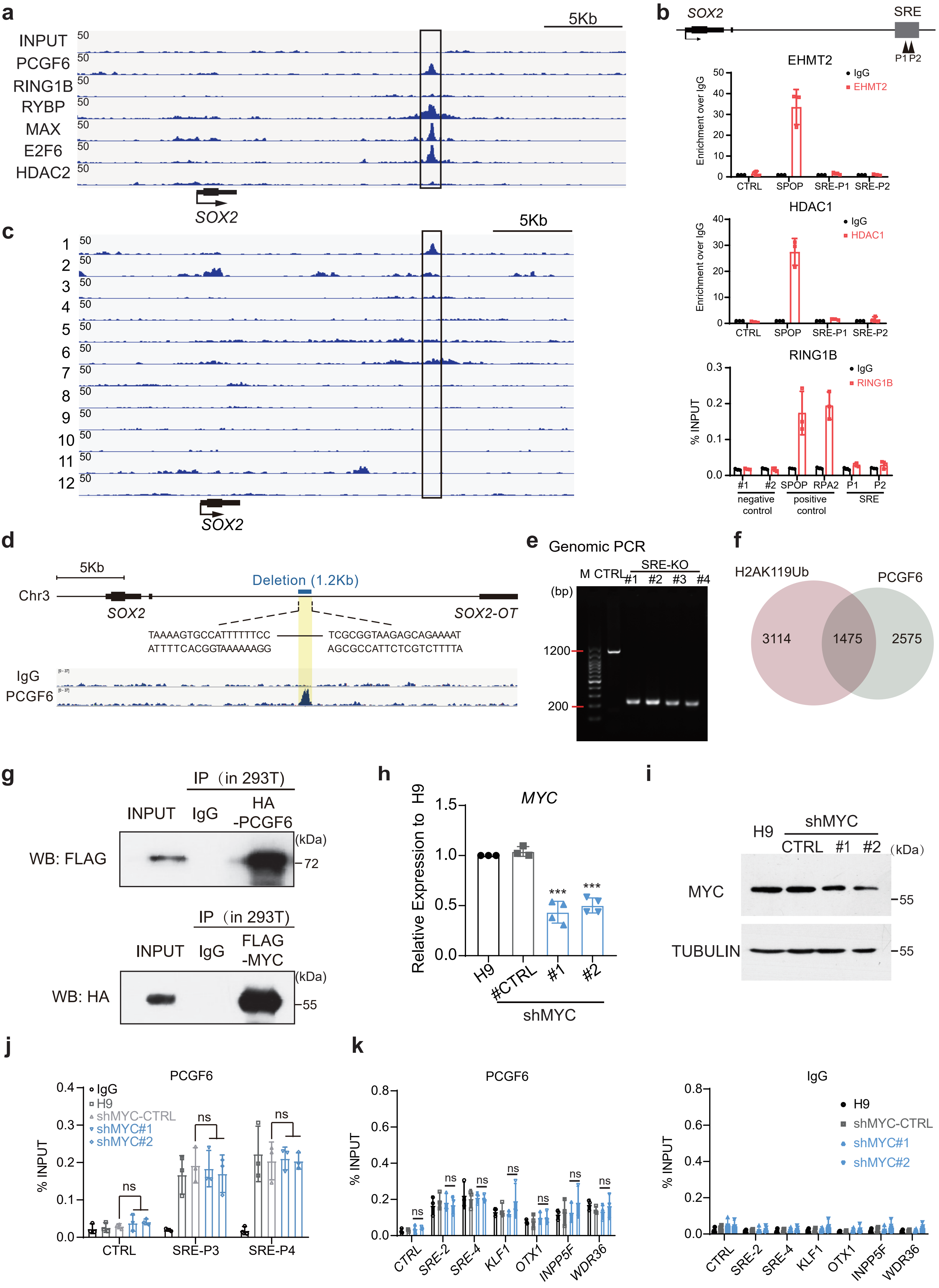
a
5Kb
INPUT
PCGF6
RING1B
RYBP
MAX
E2F6
HDAC2
SOX2
b
SOX2
SRE
P1 P2
EHMT2
IgG
EHMT2
Enrichment over IgG
CTRL
SPOP
SRE-P1
SRE-P2
HDAC1
IgG
HDAC1
RING1B
IgG
RING1B
% INPUT
#1
#2
SPOP
RPA2
P1
P2
negative control
positive control
SRE
c
5Kb
SOX2
d
5Kb
Deletion (1.2Kb)
Chr3
SOX2
SOX2-OT
TAAAAGTGCCATTTTTTCC
ATTTTCACGGTAAAAAAGG
TCGCGGTAAGAGCAGAAAAT
AGCGCCATTCTCGTCTTTTA
IgG
PCGF6
e
Genomic PCR
M CTRL
SRE-KO
#1 #2 #3 #4
(bp)
1200
200
f
H2AK119Ub
PCGF6
3114
1475
2575
g
IP (in 293T)
INPUT
IgG
HA -PCGF6
(kDa)
WB: FLAG
72
IP (in 293T)
INPUT
IgG
FLAG -MYC
(kDa)
WB: HA
55
h
MYC
Relative Expression to H9
H9
#CTRL
#1
#2
shMYC
***
i
H9
shMYC
CTRL
#1
#2
(kDa)
MYC
55
TUBULIN
55
j
PCGF6
IgG
H9
shMYC-CTRL
shMYC#1
shMYC#2
% INPUT
ns
CTRL
SRE-P3
SRE-P4
k
PCGF6
% INPUT
CTRL
SRE-2
SRE-4
KLF1
OTX1
INPP5F
WDR36
IgG
H9
shMYC-CTRL
shMYC#1
shMYC#2

Supplementary Figure 4. SRE-deficiency blocks neuroectoderm differentiation of human PSCs.

a. Bedgraph for PCGF6, RING1B, RYBP, MAX, E2F6 and HDAC2 bound at *SOX2* locus. The x axis corresponds to genomic locations with the scale indicated at the top of panel, and the y axis corresponds to ChIP-seq signal intensity. **b.** ChIP-qPCR analysis for EHMT2, HDAC1 and RING1B binding at SRE locus in human PSCs. IgG served as negative control. (n = 3). Arrowheads represent the genomic position of qPCR primers. **c.** Bedgraph for RING1B bound at *SOX2* locus in different human cell lines. 1: PCGF6 ChIP-seq in this study; 2: H2AK119Ub ChIP-seq in this study; 3~12: RING1B ChIP-seq in human cell lines, including 3: H1 cell line (GSM2805868); 4: HUES64 cell line (GSM2805868); 5: WA09/H9 reprogramed naïve human PSCs (GSE164786); 6: WA09/H9 primed human PSCs (GSE164786); 7: Bone-marrow-derived primary human mesenchymal stem cells (hMSC) (GSE125166); 8: osteogenic cells derived from bone-marrow-derived primary human mesenchymal stem cells (hBMSC) (GSE125166); 9: ME-1 cell line (GSE128771); 10: HEK 293FT cells (GSE175673); 11: the human SCLC cell line (NCI-H1963, GSE191106); 12: human chronic myeloid leukemia cell line (K562, GSE167869). The x axis corresponds to genomic locations with the scale indicated at the top of panel, and the y axis corresponds to ChIP-seq signal intensity. **d.** Schematic diagram of guide RNA design targeting SRE. The sgRNA-targeting sequences were highlighted, and the deletion is depicted by horizontal blue box. **e.** Genotyping analysis of SRE knockout human PSCs (n = 3). **f.** Venn diagrams showing the overlap of PCGF6-activated genes and MYC-binding genes. The down-regulated PCGF6 targets means the overlap of down-regulated genes identified by RNA-seq and PCGF6 target genes identified by ChIP-seq. **g.** Co-immunoprecipitation assay in 293T cells with tagged PCGF6/MYC transfection (n = 3). **h.** qRT-PCR analysis for expression of *MYC* in the WT and MYC-knockdown human PSCs. Results are shown relative to wild-type H9 (n = 3). **(i)** Western blot analysis of MYC in control and MYC-knockdown human PSCs. α-TUBULIN served as a loading control (n = 3). **j**-**k**. ChIP-qPCR analysis of PCGF6-binding in WT and MYC-knockdown human PSCs at SRE (**j**) and other

PCGF6- and MYC-co-occupied target genes (*KLF1*, *OTX1*, *FOXO3*, *INPP5F* and *WDR36*) (**k**). ChIP enrichments are normalized to 100% input (n = 3). Each point represents a biological replicate. Data are presented as the mean ± SD. Statistical significance was determined using the unpaired, two-tailed t-test in **b**, **h**, **j**-**k** (*p<0.05, **p<0.01, ***p<0.001). Exact P values and statistical parameters are provided as a Source Data file.

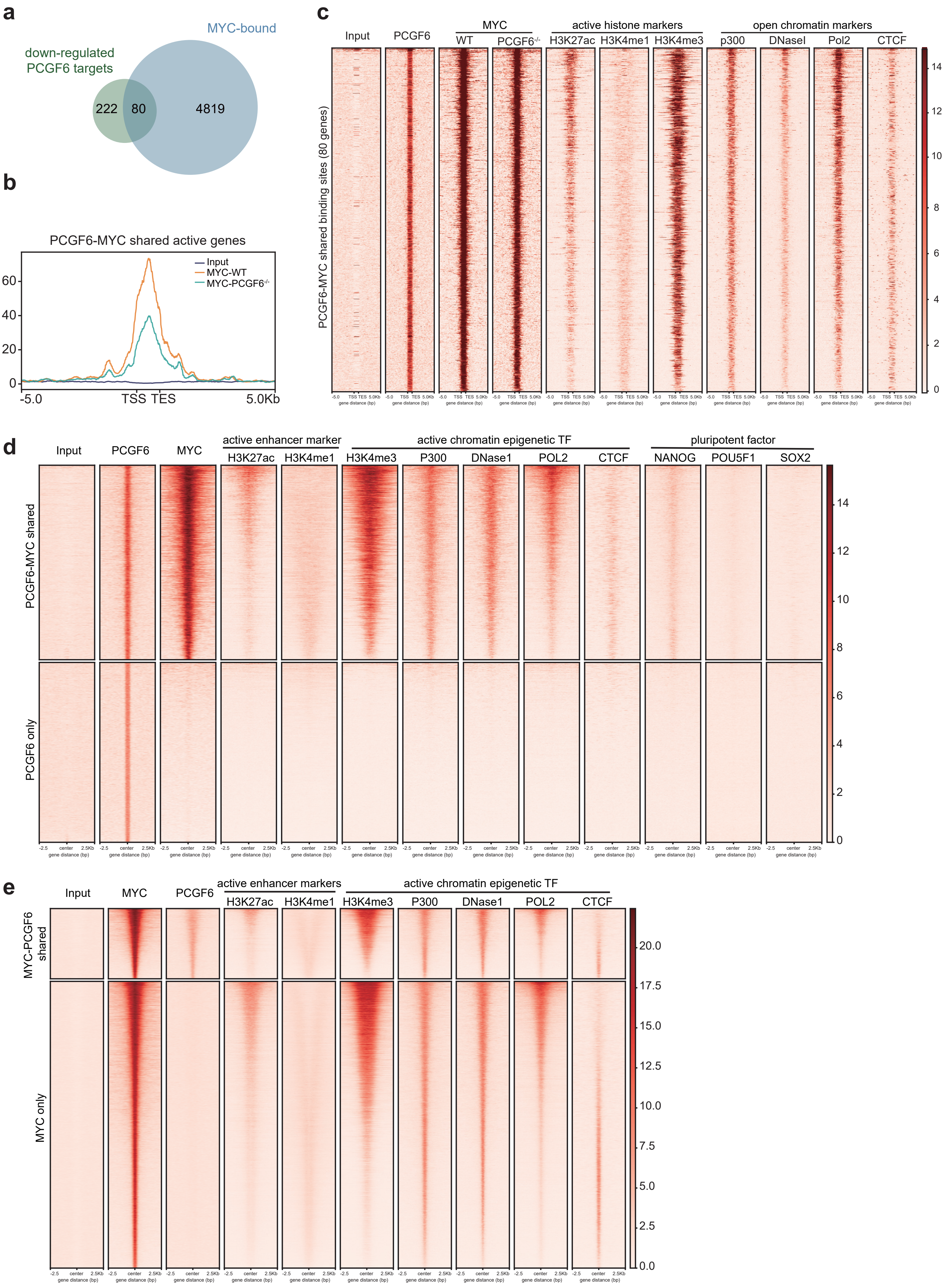

a
down-regulated PCGF6 targets
MYC-bound
222
80
4819
b
PCGF6-MYC shared active genes
Input
MYC-WT
MYC-PCGF6-/-
60
40
20
0
-5.0
TSS TES
5.0Kb
c
Input
PCGF6
MYC
WT
PCGF6-/-
active histone markers
H3K27ac
H3K4me1
H3K4me3
open chromatin markers
p300
DNaseI
Pol2
CTCF
PCGF6-MYC shared binding sites (80 genes)
14
12
10
8
6
4
2
0
d
Input
PCGF6
MYC
active enhancer marker
H3K27ac
H3K4me1
active chromatin epigenetic TF
H3K4me3
P300
DNase1
POL2
CTCF
pluripotent factor
NANOG
POU5F1
SOX2
PCGF6-MYC shared
PCGF6 only
14
12
10
8
6
4
2
0
e
Input
MYC
PCGF6
active enhancer markers
H3K27ac
H3K4me1
active chromatin epigenetic TF
H3K4me3
P300
DNase1
POL2
CTCF
MYC-PCGF6 shared
MYC only
20.0
17.5
15.0
12.5
10.0
7.5
5.0
2.5
0.0

Supplementary Figure 5. Analyses of the co-occupancy of PCGF6 and MYC.

a. The overlap of H2AK119ub binding genes and PCGF6 targets in human PSCs was displayed by Venn diagrams. The numbers showed the genes bound or co-bound by each protein identified by ChIP-seq. **b.** Tag density heatmaps illustrating changes of MYC bound in wildtype and PCGF6-KO human PSCs at PCGF6-MYC shared activated genes. **c.** Heatmaps for enrichment of PCGF6, MYC (WT and PCGF6-KO human PSCs), certain histone modifications (H3K27ac, H3K4me1 and H3K4me3) and transcription factors (P300, DNase1, Pol2 and CTCF) on PCGF6-MYC shared activated sites (±2.5 kb from the centers). ChIP-seq data were obtained from ENCODE human ESC ChIP-seq. **d.** Heatmaps for enrichment of PCGF6, MYC, certain histone modifications (H3K27ac, H3K4me1 and H3K4me3), transcription factors (P300, DNase1, Pol2 and CTCF) and pluripotent factors (NANOG, POU5F1 and SOX2) on PCGF6-MYC shared sites or PCGF6 only sites (±2.5 kb from the centers). ChIP-seq data were obtained from ENCODE human ESC ChIP-seq. **e.** Heatmaps for enrichment of PCGF6, MYC, certain histone modifications (H3K27ac, H3K4me1 and H3K4me3), transcription factors (P300, DNase1, Pol2 and CTCF) on PCGF6-MYC shared sites or MYC only sites (±2.5 kb from the centers). ChIP-seq data were obtained from ENCODE human ESC ChIP-seq.

Supplementary Table 1. Primers for qRT-PCR analyses

Primer Name	Forward Primer 5´- 3´	Reverse Primer 5´- 3´
GAPDH	AATGAAGGGGTCATTGATGG	AAGGTGAAGGTCGGAGTCAA
NANOG	CCCCAGCCTTTACTCTTCCTA	CCAGGTTGAATTGTTCCAGGTC
PCGF6	GGCCCGATCCTAGTGCG	AATAAGCGGGTAAAGACCCAGG
OCT4	CAAAGCAGAAACCCTCGTGC	TCTCACTCGGTTCTCGATACTG
SOX2	GTCATTTGCTGTGGGTGATG	AGAAAAACGAGGGAAATGGG
SOX17	GCATGACTCCGGTGTGAATCT	TCACACGTCAGGATAGTTGCAGT
FOXA2	GGAGCAGCTACTATGCAGAGC	CGTGTTCATGCCGTTCATCC
EOMES	CACATTGTAGTGGGCAGTGG	CGCCACCAAACTGAGATGAT
T	TATGAGCCTCGAATCCACATAGT	TATGAGCCTCGAATCCACATAGT
AXIN2	CTGGTGCAAAGACATAGCCA	AGTGTGAGGTCCACGGAAAC
FZD9	CGGCACCAACACAGAGAAG	CGTAGACATAGCAAACGATGAC
SYT11	AGAGGAGGATGTCATGCTAGG	GATGTAGGGGTCAGATCCCTG
NLGN3	ACAGTGGTGCTAAACCCGTC	ATTGCCATAACTGGCGAGGAT
PAX6	TCCGTTGGAACTGATGGAGT	GTTGGTATCCGGGGACTTC
SOX1	ATTATTTTGCCCGTTTTCCC	TCAAGGAAACACAATCGCTG
PDX1	TTAGGATGTGGACGTAATTCCTGTT	GGCCACTGTGCTTGTCTTCA
NKX6.1	AGACCCACTTTTTCCGGACA	CCAACGAATAGGCCAAACGA
HNF4A	ACTACATCAACGACCGCCAGT	ATCTGCTCGATCATCTGCCAG
CDX2	CTGGAGCTGGAGAAGGAGTTTC	ATTTTAACCTGCCTCTCAGAGAGC
GATA4	CAGGCGTTGCACAGATAGTG	CCCGACACCCCAATCTC
NKX2.5	CTCCCAACATGACCCTGAGT	CTCATTGCACGCTGCATAAT
TUJ1	TGGATTCGGTCCTGGATGTG	ACCTTGCTGATGAGCAACGT
MYC#1	GGCTCCTGGCAAAAGGTCA	CTGCGTAGTTGTGCTGATGT
MYC#2	TCCCTCCACTCGGAAGGAC	CTGGTGCATTTTCGGTTGTTG

Supplementary Table 2. Primers for ChIP-qPCR analyses

Primer Name	Forward Primer 5´- 3´	Reverse Primer 5´- 3´
unbound control-1	CAGACCAGCACTTTCGGTGT	TTGTCCCCAAACAAACCACC
unbound control-2	AGCTCAGGCCTCAAGACCTT	AAGAAGATGCGGCTGACTGT
SRE-P1	TCATCCTCTTCAGGCAGCATT	ACCAATATGGTGACCGTCTGAG
SRE-P2	GGTGGAAGCGTTTGTCTGATG	GGCGTTTCGAAGGCACAG
SRE-P3	TATTTAGCAAGCCGGGACGG	GGAAGGGGTGTGGTTACCG
SRE-P4	GAAACGAAAAAGCTCGCGGT	TAACCGGCTATCGGTGATGC
SRE-P5	TGACCCGACTGTGGGTACTT	AAATCAGCTGAAACCCGCCT
