# Supplementary material for: PCGF6 controls neuroectoderm specification of human pluripotent stem cells by activating SOX2 expression
Source: Nat Commun. 2022 Aug 6;13:4601. doi: 10.1038/s41467-022-32295-z (PMC9357003; doi:10.1038/s41467-022-32295-z)
Supplement: Supplementary file 3 — Description of Additional Supplementary Files [file 41467_2022_32295_MOESM3_ESM.pdf]

Description of Additional Supplementary Files

File Name: Supplementary Data 1
Description: Differentially expressed genes upon PCGF6 KO

File Name: Supplementary Data 2
Description: PCGF6 bound genes and MYC bound genes
