# Supplementary material for: PCGF6 controls neuroectoderm specification of human pluripotent stem cells by activating SOX2 expression
Source: Nat Commun. 2022 Aug 6;13:4601. doi: 10.1038/s41467-022-32295-z (PMC9357003; doi:10.1038/s41467-022-32295-z)
Supplement: Supplementary file 6 — Reporting Summary [file 41467_2022_32295_MOESM6_ESM.pdf]

nature portfolio

Corresponding author(s): Wei Jiang

Last updated by author(s): Jul 18, 2022

Reporting Summary

Nature Portfolio wishes to improve the reproducibility of the work that we publish. This form provides structure for consistency and transparency in reporting. For further information on Nature Portfolio policies, see our Editorial Policies and the Editorial Policy Checklist.

Statistics

For all statistical analyses, confirm that the following items are present in the figure legend, table legend, main text, or Methods section.

n/a	Confirmed	
☐	☒	The exact sample size (*n*) for each experimental group/condition, given as a discrete number and unit of measurement
☐	☒	A statement on whether measurements were taken from distinct samples or whether the same sample was measured repeatedly
☐	☒	The statistical test(s) used AND whether they are one- or two-sided<br>*Only common tests should be described solely by name; describe more complex techniques in the Methods section.*
☐	☒	A description of all covariates tested
☐	☒	A description of any assumptions or corrections, such as tests of normality and adjustment for multiple comparisons
☐	☒	A full description of the statistical parameters including central tendency (e.g. means) or other basic estimates (e.g. regression coefficient) AND variation (e.g. standard deviation) or associated estimates of uncertainty (e.g. confidence intervals)
☐	☒	For null hypothesis testing, the test statistic (e.g. *F*, *t*, *r*) with confidence intervals, effect sizes, degrees of freedom and *P* value noted<br>*Give P values as exact values whenever suitable.*
☒	☐	For Bayesian analysis, information on the choice of priors and Markov chain Monte Carlo settings
☒	☐	For hierarchical and complex designs, identification of the appropriate level for tests and full reporting of outcomes
☒	☐	Estimates of effect sizes (e.g. Cohen's *d*, Pearson's *r*), indicating how they were calculated

Our web collection on statistics for biologists contains articles on many of the points above.

Software and code

Policy information about availability of computer code

Data collection	Olympus IX53 for Immunostaining images. C1000 Touch Thermal Cycler machine (Bio-Rad) for qPCR. Beckman Cytoflex for Flow cytometry. MD SpectraMax i3x for MTT.
Data analysis	GraphPad Prism 8 was used for graphs and statistics. ImageJ was used for quantification of signals in western blot. FlowJo was used to analyze FACS data. Adobe Illustrator CC was used to prepare figures.<br>Hisat2 (2.1.0) was used for alignment RNA-seq data. Featurecounts (1.6.3) was used for raw counts.DEseq2 was used for finding differential genes. DAVID (https://david.ncifcrf.gov/) was used for GO analysis. GSEA was used for enrichment analysis. bowtie2 (2.3.4.3) was used for alignment CHIP-seq data. MASC2 (2.2.6) was used for peak calling. clusterProfiler (4.0.2) was used for peak annotation. Deeptools (3.3.0) was used for peak signal visualization.

For manuscripts utilizing custom algorithms or software that are central to the research but not yet described in published literature, software must be made available to editors and reviewers. We strongly encourage code deposition in a community repository (e.g. GitHub). See the Nature Portfolio guidelines for submitting code & software for further information.

Data

Policy information about availability of data

All manuscripts must include a data availability statement. This statement should provide the following information, where applicable:

- Accession codes, unique identifiers, or web links for publicly available datasets
- A description of any restrictions on data availability
- For clinical datasets or third party data, please ensure that the statement adheres to our policy

All the RNA-seq and ChIP-seq raw data have been deposited in the Gene Expression Omnibus and the accession number for the reported in this work is GSE173690. Enter token ilqxmqwenfablgf to visit the data.

Field-specific reporting

Please select the one below that is the best fit for your research. If you are not sure, read the appropriate sections before making your selection.

☒ Life sciences ☐ Behavioural & social sciences ☐ Ecological, evolutionary & environmental sciences

For a reference copy of the document with all sections, see nature.com/documents/nr-reporting-summary-flat.pdf

Life sciences study design

All studies must disclose on these points even when the disclosure is negative.

Sample size	The number of samples (at least three independent biological replicates) used in each experiment is based on previous experience.
Data exclusions	No data were excluded.
Replication	The results were reproduced at least three times as independent biological replicates.
Randomization	n/a
Blinding	The study involved unbiased quantification and analysis for immunostaining, and gene expression data sets. There was no expected outcome prior to the analysis, and blinding is not relevant.

Reporting for specific materials, systems and methods

We require information from authors about some types of materials, experimental systems and methods used in many studies. Here, indicate whether each material, system or method listed is relevant to your study. If you are not sure if a list item applies to your research, read the appropriate section before selecting a response.

Materials & experimental systems

n/a	Involved in the study	
☐	☒	Antibodies
☐	☒	Eukaryotic cell lines
☒	☐	Palaeontology and archaeology
☒	☐	Animals and other organisms
☒	☐	Human research participants
☒	☐	Clinical data
☒	☐	Dual use research of concern

Methods

n/a	Involved in the study	
☐	☒	ChIP-seq
☐	☒	Flow cytometry
☒	☐	MRI-based neuroimaging

Antibodies

Antibodies used

PCGF6 (1:1000, Proteintech, Cat#24102-1-AP), GAPDH (1:5000, Proteintech, Cat#60004-l-lg), α-Tubulin (1:5000, Proteintech, Cat#11224-1-AP), OCT4 (1:1000, Santa Cruz Biotechnology, Cat#sc-5279), SOX2 (1:100, Santa Cruz Biotechnology, Cat#sc-20088), NANOG (1:1000, Santa Cruz Biotechnology, Cat#sc-33759), MYC (1:1000, Cell Signaling Technology, Cat#13987S), PDX1 1:200, abcam, Cat#ab47267), T/Brachyury (1:200, Cell Signaling Technology, Cat#81694S), TUJ1 (1:200, SIGMA, Cat#T3952) were used in western blot.
PAX6 (1:200, Biolegend, Cat#l901301), SOX1 (1:200, Cell Signaling Technology, Cat#4195S), OCT4 (1:200, Santa Cruz biotechnology, Cat#sc-5279), NANOG (1:200, Cell Signaling Technology, Cat#4903), MYC (Cell Signaling Technology, Cat#13987S), RING1B (Cell Signaling Technology, Cat#5694) were used in immunofluorescence assay.
APC-conjugated mouse-anti-human CD184 (BD, Cat#555976) and APC-conjugated mouse isotype IgG (BioLegend, Cat# 400220) were used in flow cytometry analysis.

	PCGF6 (1:200, Proteintech, Cat#24102-1-AP), H2AK119Ub (1:200, Cell Signaling Technology, Cat#8240), MYC (Cell Signaling Technology, Cat#13987S) were used in ChIP-seq.
Validation	PCGF6 (1:1000, Proteintech, Cat#24102-1-AP) was validated by our Western Blot experiment with WT and PCGF6-KO cell lines; GAPDH (1:5000, Proteintech, Cat#60004-l-lg), α-Tubulin (1:5000, Proteintech, Cat#11224-1-AP), OCT4 (1:1000, Santa Cruz Biotechnology, Cat#sc-5279), SOX2 (1:100, Santa Cruz Biotechnology, Cat#sc-20088), NANOG (1:1000, Santa Cruz Biotechnology, Cat#sc-33759), MYC (1:1000, Cell Signaling Technology, Cat#13987S),PAX6 (1:200, Biolegend, Cat#l901301), SOX1 (1:200, Cell Signaling Technology, Cat#4195S), APC-conjugated mouse-anti-human CD184 (BD, Cat#555976), APC-conjugated mouse isotype IgG (BioLegend, Cat# 400220),H2AK119Ub (1:200, Cell Signaling Technology, Cat#8240),PDX1 1:200, abcam, Cat#ab47267), T/Brachyury (1:200, Cell Signaling Technology, Cat#81694S), TUJ1 (1:200, SIGMA, Cat#T3952) MYC (Cell Signaling Technology, Cat#13987S), RING1B (Cell Signaling Technology, Cat#5694) were validated on the manufacturer's website.

Eukaryotic cell lines

Policy information about cell lines

Cell line source(s)	Human iPSC line PGP1 is described in Wang, G. et al (2014) and referred in manuscript. H9 is from WiCell.
Authentication	Routine quality control by microscopy morphology, Immunostaining for pluripotent markers, as well as EB assay.
Mycoplasma contamination	All cell lines were confirmed as mycoplasma negative before experiments.
Commonly misidentified lines (See ICLAC register)	No commonly misidentified line was used.

ChIP-seq

Data deposition

☒ Confirm that both raw and final processed data have been deposited in a public database such as GEO.

☒ Confirm that you have deposited or provided access to graph files (e.g. BED files) for the called peaks.

Data access links *May remain private before publication.*	To review GEO accession GSE173690: Go to https://www.ncbi.nlm.nih.gov/geo/query/acc.cgi?acc=GSE173690<br>Enter token ilqxmqwenfablgf to visit the data.
Files in database submission	Input1_1_R1.fq.gz<br>Input1_1_R2.fq.gz<br>Input2_1_R1.fq.gz<br>Input2_1_R2.fq.gz<br>PCGF6_1_R1.fq.gz<br>PCGF6_1_R2.fq.gz<br>PCGF6_2_R1.fq.gz<br>PCGF6_2_R2.fq.gz<br>Ub-WT-1_R1.fq.gz<br>Ub-WT-1_R2.fq.gz<br>Ub-KO1-1_R1.fq.gz<br>Ub-KO1-1_R2.fq.gz<br>E2F6_R1.fastq.gz<br>E2F6_R2.fastq.gz<br>MAX_R1.fastq.gz<br>MAX_R2.fastq.gz<br>KO_E2F6_R1.fastq.gz<br>KO_E2F6_R2.fastq.gz<br>KO_MAX_R1.fastq.gz<br>KO_MAX_R2.fastq.gz<br>MYC_R1.fastq.gz<br>MYC_R2.fastq.gz<br>KO_MYC_R1.fastq.gz<br>KO_MYC_R2.fastq.gz
Genome browser session (e.g. UCSC)	BW files were visualized in IGV and the reference genome assembly was Human hg38.

Methodology

Replicates	The results were reproduced twice as experimental replicates.
Sequencing depth	Input1_1_R1.fq.gz:total numbers-15452581; uniquely reads-13565821; length of reads-150; paired-end.<br>Input1_1_R2.fq.gz:total numbers-15452581; uniquely reads-13565821; length of reads-150; paired-end.<br>PCGF6_1_R1.fq.gz:total numbers-23361005; uniquely reads-20508626; length of reads-150; paired-end.<br>PCGF6_1_R2.fq.gz:total numbers-23361005; uniquely reads-20508626; length of reads-150; paired-end.<br>Input2_1_R1.fq.gz, Input2_1_R2.fq.gz, PCGF6_2_R1.fq.gz, PCGF6_2_R2.fq.gz, Ub-WT-1_R1.fq.gz, Ub-WT-1_R2.fq.gz, Ub-KO1-1_R1.fq.gz, Ub-KO1-1_R2.fq.gz,2F6_R1.fastq.gz, E2F6_R2.fastq.gz, MAX_R1.fastq.gz, MAX_R2.fastq.gz, KO_E2F6_R1.fastq.gz,

	KO_E2F6_R2.fastq.gz, KO_MAX_R1.fastq.gz, KO_MAX_R2.fastq.gz, MYC_R1.fastq.gz, MYC_R2.fastq.gz, KO_MYC_R1.fastq.gz, KO_MYC_R2.fastq.gz: total numbers- at least 15M; uniquely reads-at least 10M; length of reads-150; paired-end.
Antibodies	PCGF6 (1:200, Proteintech, Cat#24102-1-AP), H2AK119Ub (1:200, Cell Signaling Technology, Cat#8240), MYC (1:1000, Cell Signaling Technology, Cat#13987S) were used in ChIP-seq.
Peak calling parameters	nohup bowtie2 -p 30 -x /data/Jlab-ds/index/hg38_bowtie2/hg38 -1 *.fastq.gz -2 *.fastq.gz | samtools view -bS | samtools sort -o *.bam &<br>nohup macs2 callpeak -t input.bam -c *.bam -f BAMPE -B -p 0.005 -g hs -n input --outdir ./callpeaks/ &
Data quality	All peaks were filtered by the parameters (p<0.005, fold-enrichment >2).
Software	reads were qualified by FastQC (v0.11.9) and the adapter was trimmed by Trim galore (v0.6.2). Then these clean reads were aligned to the human reference genome GRCh38 using Bowtie2 (v2.3.4.2) with end-to-end. The uniquely mapped reads were sorted by Samtools (v1.9) and then performed peak calling by MACS2 (v2.1.2) with the callpeak module (the parameter --call-summits --extsize 150 -p 0.05).The most significantly enriched peaks were selected with a threshold by corrected Foldchange > 3. The bam files were converted to the bigWig signal files by DeepTools (v3.3.0) with the bamCoverage module and then visualized using the DeepTools module of computeMatrix, plotHeatmap and plotProfile. Homer was used to annotated nearby genes from the peaks obtained from MACS2 and to find motif that enrich in the binding sequence. Gene ontology analysis was performed on DAVID (https://david.ncifcrf.gov).

Flow Cytometry

Plots

Confirm that:

☒ The axis labels state the marker and fluorochrome used (e.g. CD4-FITC).

☒ The axis scales are clearly visible. Include numbers along axes only for bottom left plot of group (a 'group' is an analysis of identical markers).

☒ All plots are contour plots with outliers or pseudocolor plots.

☒ A numerical value for number of cells or percentage (with statistics) is provided.

Methodology

Sample preparation	TrypLE Express (Gibco, Cat#12604013) was used to detach cultured cells. Then the cells were washed twice with DPBS containing 2% FBS and incubated with APC-conjugated mouse-anti-human CD184 (BD, Cat#555976) or APC-conjugated mouse isotype IgG (BioLegend, Cat# 400220) in dark for 30 minutes at 4°C. After washing twice, the cells were resuspended by DPBS.
Instrument	CytoFLEX flow cytometer (Beckman)
Software	FlowJo
Cell population abundance	10000 cells exclude cell debris.
Gating strategy	Cell debris was excluded using a FSC vs SSC gate; aggregates were excluded via a SSC-H vs SSC-A approach.

☒ Tick this box to confirm that a figure exemplifying the gating strategy is provided in the Supplementary Information.
